# Effect of Solution Treatment on the Shape Memory Functions of (TiZrHf)_50_Ni_25_Co_10_Cu_15_ High Entropy Shape Memory Alloy

**DOI:** 10.3390/e21101027

**Published:** 2019-10-22

**Authors:** Hao-Chen Lee, Yue-Jin Chen, Chih-Hsuan Chen

**Affiliations:** 1 Department of Mechanical Engineering, National Taiwan University, Taipei 106, Taiwan; 2 Department of Materials Science and Engineering, National Taiwan University, Taipei 106, Taiwan

**Keywords:** high entropy alloy, shape memory alloy, shape memory effect, pseudoelasticity, martensitic transformation

## Abstract

This study investigated the effects of solution treatment at 1000 °C on the transformation behaviors, microstructure, and shape memory functions of a novel (TiZrHf)_50_Ni_25_Co_10_Cu_15_ high entropy shape memory alloy (HESMA). The solution treatment caused partial dissolution of non-oxygen-stabilized Ti_2_Ni-like phase. This phenomenon resulted in the increment of (Ti, Zr, Hf) content in the matrix and thus increment of the M_s_ and A_f_ temperatures. At the same time, the solution treatment induced a high entropy effect and thus increased the degree of lattice distortion, which led to increment of the friction force during martensitic transformation, resulting in a broad transformation temperature range. The dissolution of the Ti_2_Ni-like phase also improved the functional performance of the HESMA by reducing its brittleness and increasing its strength. The experimental results presented in this study demonstrate that solution treatment is an effective and essential way to improve the functional performance of the HESMA.

## 1. Introduction

Since the new metallurgical concept of high entropy alloys (HEAs) was proposed by Yeh et al. [1], extensive efforts have been made to explore undiscovered alloys with multiple compositions. These alloys with complex compositions are also known as complex concentrated alloys (CCAs) or multi-principal element alloys (MPEAs), terms which reflect the high quantities of alloying elements they contain. With the aid of this new alloy design concept, numerous alloy systems with unique properties have been found and investigated, some examples being CoCrFeMnNi, which has extraordinary ductility at low temperature [2]; ZrNbTiVHf, with a single BCC phase [3]; and VCoNi, with ultrastrong mechanical properties [4]. It is noted that most investigations on HEAs have concentrated on structural materials for applications at low-temperature [2] or in refractory environments [5], bioapplications [6], or for applications requiring a great combination of strength and ductility [7,8]. In recent years, studies on HEAs have extended to non-equiatomic compositions for better desired or unique properties, such as light-weight [9], high phase stability [10], outstanding radiation resistance [11], etc. Assocaited with these newly explored properties, interpretations of fundamental mechanisms in HEAs have been investigated with both experimental and simulation techniques [12,13,14,15,16]. With the understanding of the atomic distribution and deformation mechanisms, the design and research progress of HEAs can step further [17]. These studies provide new aspects and demonstrate the potentials for developing novel materials with performance surpassing the properties of materials now served in our daily life. As a consequence, developing HEAs with different functionalities to serve as next-generation materials is the main aim of the research society.

Recently, functional HEAs with shape memory characteristics have attracted attention from researchers worldwide. The concept of the high entropy shape memory alloy (HESMA) was first published by Firstov et al. [18,19,20]. They designed HESMAs by adding Zr and Hf to substitute for the Ti atoms and Co and Cu to substitute for the Ni atoms in the well-known binary TiNi shape memory alloy (SMA). They first demonstrated that Ti_16.67_Zr_16.67_Hf_16.67_Ni_25_Cu_25_ is able to undergo thermoelastic martensitic transformation with a M_s_ (martensitic transformation starting temperature) and A_f_ (austenitic transformation finishing temperature) of 226 °C and 338 °C, respectively [19]. Afterward, they proposed that HESMAs have high potential to be applied as high temperature SMAs [20]. Recently, Canadinc et al. reported multi-component SMAs with ultra-high transformation temperatures; for instance, the A_s_ of Ni_25_Pd_25_Ti_16.67_Hf_16.67_Zr_16.67_ reached nearly 800 °C [21]. They also demonstrated that Ni_35_Pd_15_Ti_30_Hf_20_ and Ni_35_Pd_15_Ti_30_Hf_20_ exhibit superelastic behavior at 750 °C. Wang et al. reported Ti-rich high entropy alloys that undergo stress-induced β to α” martensitic transformation [22]. This martensitic transformation is reversible and can contribute well-defined superelastic behavior with recoverable strain of up to 5.2%. The present authors demonstrated that the Ti_16.67_Zr_16.67_Hf_16.67_Ni_25_Co_10_Cu_15_ alloy is a HESMA with thermoelastic martensitic transformation [23]. This alloy is based on the binary TiNi SMA, which showed high solubility of Zr, Hf, Co, and Cu atoms [24]. In this alloy, Zr and Hf were added to substitute for Ti; Co and Cu were added to substitute for Ni. The Ti_16.67_Zr_16.67_Hf_16.67_Ni_25_Co_10_Cu_15_ HESMA undergoes B2 to B19′ martensitic transformation, which is the same as that of the binary TiNi SMA. The Ti_16.67_Zr_16.67_Hf_16.67_Ni_25_Co_10_Cu_15_ HESMA shows a recoverable strain of 4.8% under 650 MPa, which is comparable to that of a conventional TiNi alloy under 200 MPa (4.9%). These recent studies confirm that the concept of the high entropy alloy can be utilized to develop new SMAs that exhibit higher strength or can be applied at higher application temperatures. In this study, we found that solution treatment of Ti_16.67_Zr_16.67_Hf_16.67_Ni_25_Co_10_Cu_15_ HESMA causes an increment in transformation temperature and also improves its shape memory functions.

## 2. Materials and Methods

The Ti_16.67_Zr_16.67_Hf_16.67_Ni_25_Co_10_Cu_15_ HESMA ingot was prepared with a vacuum arc remelter (VAR). High-purity raw materials of Ti (>99.99 wt%), Zr (>99.95 wt%), Hf (>99.9 wt%), Ni (>99.99 wt%), Co (>99.9 wt%), and Cu (>99.99 wt%) were weighed and melted to fabricate the ingot. The chamber was first evacuated to vacuum pressure of 10^−2^ torr and then feedbacked with high-purity Ar (99.9999%) to a pressure of 300 torrs. The evacuation and feedback procedures were repeated six times to reduce the oxygen content in the chamber. Finally, the Ar pressure of 300 torrs was remained in the chamber to trigger the electric arc during the VAR process. The solidified ingot was homogenized under vacuum at 900 °C for 24 h, and then at 950 °C for 12 h, followed by furnace cooling. The specimen in the furnace-cooled condition is referred to as FC in the following paragraphs. The FC ingot was sliced into pieces and sealed in a quartz tube for solution treatment. The quartz tube was first evacuated to a pressure below 10^−2^ torr and feedbacked with Ar. The sealed specimens were solution treated at 1000 °C for 2 h, followed by water quenching. The water-quenched sample is referred to as WQ in this text. The transformation temperatures of the FC and WQ samples were measured with a differential scanning calorimeter (DSC, DSC 25, TA Instruments, New Castle, DE, USA). The cooling and heating rates were 10 °C/min. The phase constitutions of the HESMAs were measured with a high-power monochromatized X-ray diffractometer (XRD, TTRAX III, Rigaku Co., Tokyo, Japan). Cu K_α_ was used to collect the diffraction information with a scanning rate of 4°/min. The compositions of the FC and WQ samples were measured with an electron probe microanalyzer (EMPA, JXA-8200, JEOL, Tokyo, Japan). The composition was determined by averaging 10 randomly measured points. The microstructures of the Ti_16.67_Zr_16.67_Hf_16.67_Ni_25_Co_10_Cu_15_ HESMAs were observed with a scanning electron microscope (SEM, Nova 650, FEI, Hillsboro, Oregon, United States) and a transmission electron microscope (TEM, Tecnai™ G2 F30, FEI, Hillsboro, Oregon, United States) operated at 300 kV. The shape memory effects of the HESMAs were tested with a dynamic mechanical analyzer (DMA, DMA 2980, TA Instruments, New Castle, DE, USA). The samples were tested in a three-point bending mode, and the spacing of the supporting pins was 20 mm. The sample was first heated to 150 °C (above A_f_) and then loaded with constant flexural stress. The sample was then cooled to −100 °C and heated back to 150 °C with cooling and heating rates of 3 °C/min. During the cooling/heating cycle, constant flexural stress was applied. After each cycle, the subsequent cooling/heating run was started with increased flexural stress. The pseudoelasticity of the HESMAs was tested with a universal tester equipped with a 50 kg load cell (AG-IS 50KN, Shimadzu, Kyoto, Japan). A TEC-N300 environmental furnace was utilized to control the testing temperature at 150 °C. The specimens were compressed with successively increasing applied strain. The strains of the specimens were measured and recorded with a digital image correlation method (VIC-gauge, Correlated Solutions Inc., Columbia, SC, USA).

## 3. Results and Discussions

### 3.1. Transformation Behaviors

**Figure 1:**
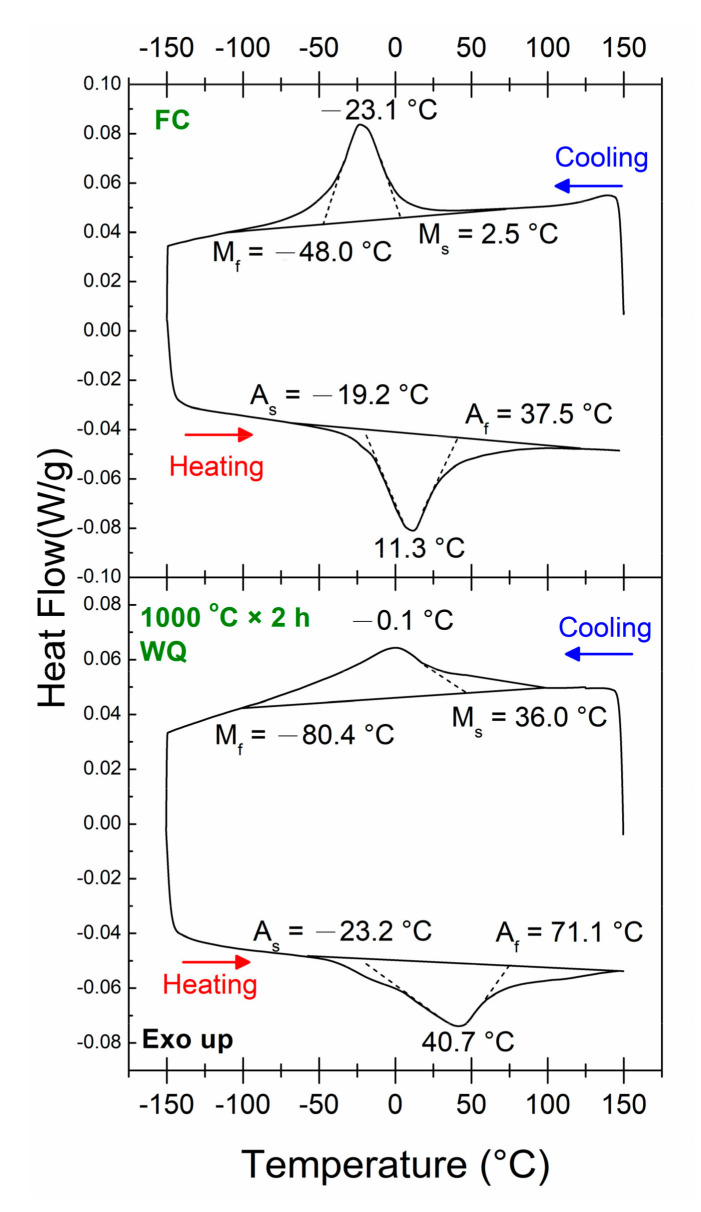
The heat flow curves of the furnace-cooled (FC) and water-quenched (WQ) Ti_16.67_Zr_16.67_Hf_16.67_Ni_25_Co_10_Cu_15_ HESMAs measured with DSC.

**Figure 2:**
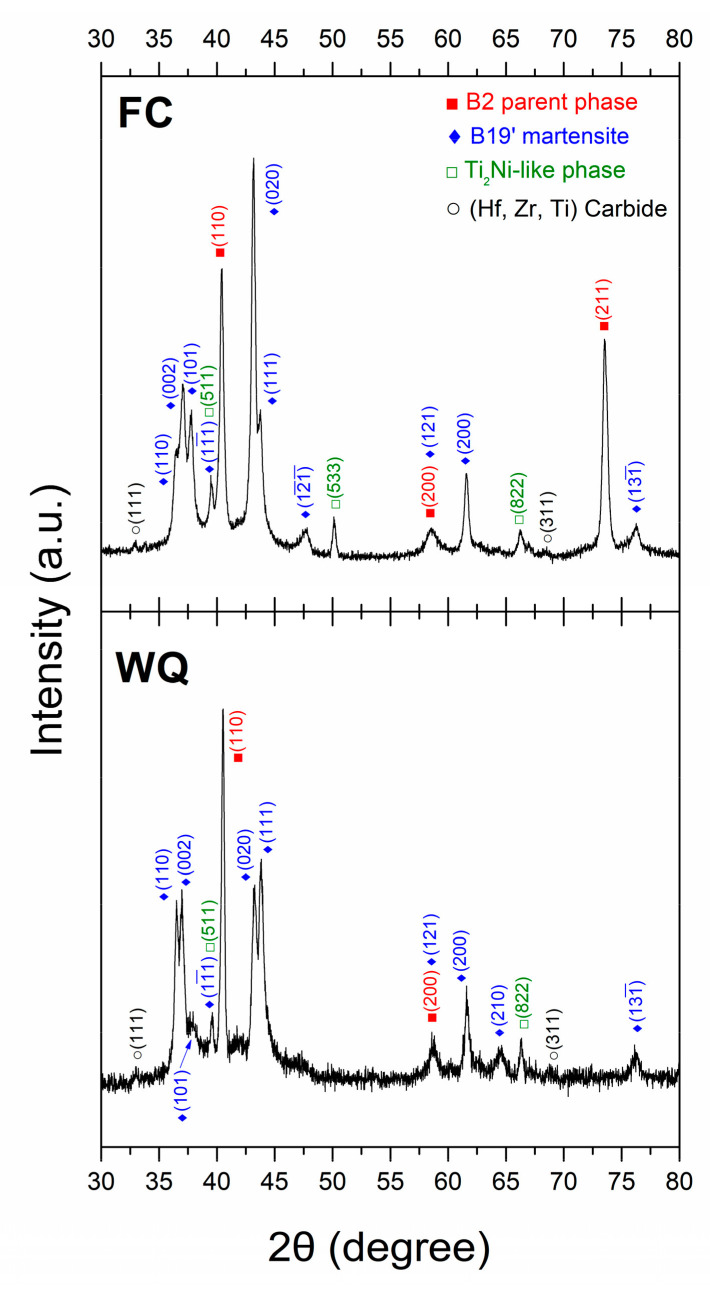
XRD spectra of the FC and WQ Ti_16.67_Zr_16.67_Hf_16.67_Ni_25_Co_10_Cu_15_ HESMAs collected at room temperature.

The DSC heat flow curves of the FC and WQ Ti_16.67_Zr_16.67_Hf_16.67_Ni_25_Co_10_Cu_15_ HESMAs are shown in Figure 1. The transformation temperatures of these alloys were determined by tangent method. As can be seen in Figure 1, both the FC and WQ samples underwent a one-stage martensitic transformation during cooling and heating. It is noted that the WQ sample exhibited broad transformation peaks during both cooling and heating. The M_s_, M_p_ (martensitic transformation peak temperature), A_p_ and A_f_ (austenitic transformation peak temperature) of the WQ sample were higher than those of the FC one. On the other hand, the M_f_ (martensitic transformation finishing temperature) and A_s_ (austenitic transformation starting temperature) of the WQ sample were lower. Since the transformation temperature of a SMA is significantly sensitive to its composition [25] and internal stress [26,27], these changes in transformation temperature imply that the composition of the B2 matrix was altered during the solution treatment. Associated with the changes in transformation temperatures, the amounts of martensite at room temperature increased. The fraction of B2 matrix that has transformed to martensite was estimated by the area fraction of the DSC peak (cooling) above 25 °C. The results showed that the fraction of the martensite at RT increased from 3.4 to 21.9% due to the increment of transformation temperatures after the solution treatment. Figure 2 shows the XRD spectra of FC and WQ samples collected at room temperature. Both samples contained B2 parent phase and B19′ martensite at room temperature, as well as some Ti_2_Ni-like second phase. Ti_2_Ni-like second phase was also identified in a similar HESMA [21]. The XRD spectra also demonstrated that the FC and WQ Ti_16.67_Zr_16.67_Hf_16.67_Ni_25_Co_10_Cu_15_ HESMAs both underwent B2 to B19′ martensitic transformation. Some small diffraction peaks originated from some (Ti, Zr, Hf) carbides were also identified. The carbide phase has been reported in the previous study that it formed due to some impurities in the raw materials [23].

### 3.2. Microstructure Observations

**Figure 3:**
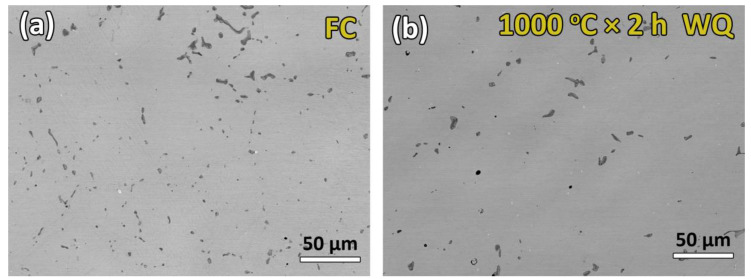
SEM backscattered electron image (BSI) of (**a**) FC (**b**) WQ Ti_16.67_Zr_16.67_Hf_16.67_Ni_25_Co_10_Cu_15_ HESMAs.

Figure 3a,b presents SEM backscattering electron (BSE) images of the FC and WQ Ti_16.67_Zr_16.67_Hf_16.67_Ni_25_Co_10_Cu_15_ HESMAs, respectively. The FC and WQ samples were both composed of grey B2 matrix and dark Ti_2_Ni-like precipitates. Additionally, some small (Ti, Zr, Hf) carbide particles were observed in white contrast. The area fractions of the carbide phase in the FC and WQ samples in Figure 3a,b were estimated to be 0.05% and 0.04% for the FC and WQ samples, respectively. The compositions of the B2 matrix and Ti_2_Ni-like phase are listed in Table 1. As can be seen in Table 1, the composition of the dark precipitates was close to (TiZrHf)_2_(NiCoCu) and was identified as the Ti_2_Ni-like phase frequently observed in conventional TiNi-based SMAs. Additionally, the Ti_2_Ni-like phase is usually stabilized by oxygen [28] to form Ti_4_Ni_2_O_x_ phase; therefore, the O content in the Ti_2_Ni-like phase was also measured and is shown in Table 1. It can be clearly seen from Table 1 that the O content in the Ti_2_Ni-like phase significantly increased after the solution treatment. The accumulated oxygen may have originated from the trace oxidation of the raw materials or the low vacuum level during the melting and heat treatment processes. It is also noted that the volume fraction of the Ti_2_Ni-like phase decreased and the size of the precipitates increased after the solution treatment, as shown in Figure 3. The volume fractions of the Ti_2_Ni-like phase in the FC and WQ conditions were estimated by averaging the area of the Ti_2_Ni-like phase of at least six SEM images, and the results are shown in Table 2. Table 2 shows that the volume fraction of the Ti_2_Ni-like phase decreased by about 31% during the solution treatment. These features are consistent with the results in TiNi SMA reported by Kai et al. [29]. They reported that the volume fraction of Ti_2_Ni decreased and the oxygen content in the Ti_2_Ni increased during solution treatment at 1000 °C. They also showed that some Ti_2_Ni dissolved into the matrix and that the oxygen content accumulated in the oxygen-stabilized Ti_4_Ni_2_O_x_ phase, so the oxygen content in the Ti_2_Ni increased from 0.75 at.% to 2.26 at.% after the TiNi SMA was solution treated at 1000 °C for 24 h. It is noted that the grain size of the FC and WQ samples were determined with optical images to be 27.5 µm and 32.3 µm, respectively. These results show that the grain size of the (TiZrHf)_50_Ni_25_Co_10_Cu_15_ high entropy SMA did not significantly increase during the solution treatment at 1000 °C for 2 h.

On the other hand, the composition of the matrix was quite close to the nominal composition of the alloy. However, due to the formation of Ti_2_Ni-like phase, the (Ti, Zr, Hf) content in the matrix slightly decreased, so the composition of the matrix became (Ni, Co, Cu)-rich, such as (TiZrHf)_48.9_(NiCoCu)_51.1_ and (TiZrHf)_49.4_(NiCoCu)_50.6_ in the FC and WQ samples, respectively. It can be expected that since some Ti_2_Ni-like phase dissolved into the matrix during solution treatment, the (Ti, Zr, Hf) content was slightly increased from 48.9 to 49.4 at.% after solution treatment.

### 3.3. Changes in Transformation Temperatures

As shown in Figure 1, the M_s_ and A_s_ temperatures of the WQ ribbon increased after the solution treatment, meaning that the martensitic transformation was stabilized such that it occurred at a higher temperature. The possible reasons for the changes in transformation temperatures are discussed here.

First, it is noted that the addition of Zr and Hf to substitute for Ti as alloying elements increases the transformation temperatures of TiNi when their contents are higher than 10 at.% [30]. On the other hand, the addition of Co to substitute for Ni significantly decreases the transformation temperatures [31]; the addition of Cu to substitute for Ni also slightly decreases the transformation temperatures [32]. As a consequence, the increment of transformation temperatures after solution treatment implies that the Ti, Zr, or Hf content of the matrix may have increased after the solution treatment.

Second, the Ti-rich TiNi SMAs (Ti content > 50 at.%) exhibit higher transformation temperatures than do the Ni-rich ones (Ni content > 50 at.%) [28]. Therefore, the transformation temperatures increased if the (Ti, Zr, Hf) content increased after the solution treatment.

Since the variation of transformation temperature may originate from changes in composition, some second phase rich in Ti, Zr, or Hf must have been dissolved in the matrix during the solution treatment. One possible second phase is the Ti_2_Ni-like phase, as shown in Figure 3. The other possible second phase may be the nano-scale H-phase, which forms after aging of the solution-treated Ni-rich TiNiZr [33,34] or TiNiHf [35,36] high temperature SMAs.

For high entropy alloys, the sluggish diffusion feature caused by the complex atomic configuration may retard phase transformation. However, since the martensitic transformation is a diffusionless phase transformation, the sluggish diffusion nature will not retard the phase transformation from austenite to martensite. On the other hand, the stress field and changes in composition in SMAs are the main reasons for changes in transformation temperatures. As some published studies on high entropy SMAs show, whether the austenite or martensite is stabilized (decreasing or increasing in transformation temperature) is not determined by the number of alloying elements. Canadinc et al. showed that increasing Zr, Hf, or Pd content resulted in increased transformation temperature [4]. Firstov et al. showed that the addition of Co decreased the transformation temperature [20]. As a consequence, the transformation temperatures of the (TiZrHf)(NiCoCu) high entropy SMA system are considered still mainly governed by the conventional effects of elements on the TiNi SMA as discussed above, and the solution treatment will not always cause stabilization of martensite (increment in transformation temperatures) in high entropy SMAs.

As shown in Figure 3 and Table 2, the volume fraction of the Ti_2_Ni-like phase, which is rich in (Ti, Zr, Hf), decreased after solution treatment. This feature implied that some of these Ti_2_Ni-like phases exhibited lower thermal stability and dissolved into the matrix during the solution treatment, and thus increased the (Ti, Zr, Hf) content in the matrix, as can be seen in Table 1. This increment in the (Ti, Zr, Hf) content from 48.9 to 49.4 at.% contributed to the increment in transformation temperature after solution treatment.

**Figure 4:**
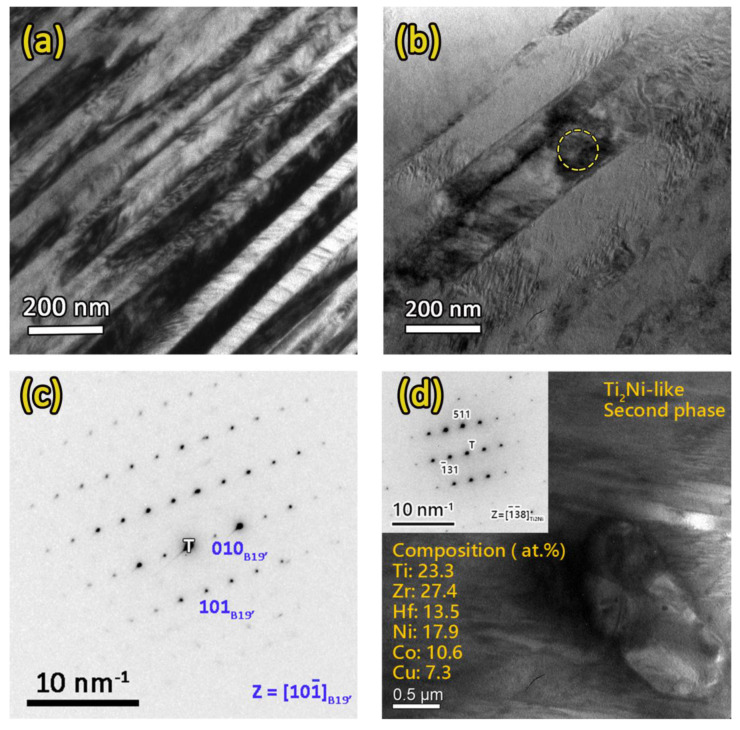
(**a**,**b**) Bright field (BF) images of the FC sample, showing a lamellar B19′ martensite structure. (**c**) The selected area diffraction pattern (SADP) taken from the circled area shown in (**b**), which shows the diffraction pattern of B19′ martensite. (**d**) A Ti_2_Ni-like phase with size of several micrometers in the matrix. The inset in (**d**) shows the diffraction pattern taken from the Ti_2_Ni-like phase.

To understand if any nano-scale precipitates existed in the FC sample, TEM observations were carried out to reveal its microstructures. Figure 4 shows the microstructures of the FC sample. In Figure 4a,b, the lamellar martensite structure of the FC samples can be observed. A selected area diffraction pattern (SADP) taken from the circled area, shown in Figure 4c, shows the diffraction pattern of B19′ martensite. It is noted that, from the observations shown in Figure 4a,b, no nano-scale precipitates were found in the matrix of the FC sample. Additionally, the SADP in Figure 4c shows the diffraction pattern of only B19′, without any other second phase. In the matrix, only Ti_2_Ni-like second phases with sizes of several micrometers could be observed, as shown in Figure 4d. The composition of the second phase was determined by energy-dispersive X-ray spectroscopy (EDS) to be Ti_23.3_Zr_27.4_Hf_13.5_Ni_17.9_Co_10.6_Cu_7.3_, which was quite close to the composition measured with EPMA, as shown in Table 1. As a consequence, this second phase was confirmed to be the Ti_2_Ni-like phase, as those observed in the SEM observations. Additionally, the SADP taken from this Ti_2_Ni-like phase is shown in the inset of Figure 4d; it had the same structure as the Ti_2_Ni but a slightly larger lattice constant of 1.187 nm [37]. The WQ sample had no nano-scale precipitates because it was solution-treated at 1000 °C. The microstructure of the WQ sample has been reported previously to have no nano-scale precipitates in the matrix [23]. Since the TEM observations revealed the absence of nano-scale precipitates in the FC sample, the increment in M_s_ temperature was not caused by the dissolution of nano-scale precipitates. According to the SEM and TEM observations and the composition analysis, the increment of the M_s_ and A_f_ transformation temperatures after solution treatment can be attributed to the partial dissolution of the Ti_2_Ni-like phase, which resulted in an increased amount of (Ti, Zr, Hf) content in the matrix.

It is also noted that, unlike the M_s_ and A_f_, the M_f_ and A_s_ temperatures of the WQ Ti_16.67_Zr_16.67_Hf_16.67_Ni_25_Co_10_Cu_15_ HESMA decreased after the solution treatment, resulting in a broad transformation peak and an increment of the transformation span, as can be seen in Figure 1. This feature implies that the martensitic transformation occurred in a much broader temperature window after solution treatment. As discussed in a previous study, the broad transformation temperature range may have originated from composition inhomogeneity and severe lattice distortion; both reasons originate from the complex composition of the senary Ti_16.67_Zr_16.67_Hf_16.67_Ni_25_Co_10_Cu_15_ HESMA. However, as can be seen from Table 1, EPMA measurements showed that the FC and WQ samples exhibited similar compositional deviations. As a consequence, compositional inhomogeneity may not have been the main cause of the broadening of the transformation range. On the other hand, as frequently noted in Ni-rich TiNi SMAs, the strain field around the Ti_3_Ni_4_ precipitates broadens the B2→B19′ transformation peak [38]. Due to the large transformation strain of B2→B19′ transformation, its transformation temperature will be significantly influenced by the strain field. For comparison, the transformation behavior of B2→R, which is associated with a much smaller transformation strain, is hardly affected by the strain field around Ti_3_Ni_4_ precipitates [26]. Since no nano-scale precipitates were observed in the FC and WQ samples, it is expected that severe lattice distortion caused local strain fields to develop. The severe lattice distortion occurred in the senary Ti_16.67_Zr_16.67_Hf_16.67_Ni_25_Co_10_Cu_15_ HESMA due to the complexity of its composition, which was reflected in its higher hardness (394 HV) than that of the TiNi SMA (229 HV). This severe lattice distortion was considered to be the origin of the broadened transformation behavior after the solution treatment. Since the FC sample experienced a slow cooling history from 950 °C, there was enough time for the ingot to undergo the diffusion process, which allowed atoms to move to positions where the lattice distortion was minimized, and the internal strain field in the alloy was thus reduced. In contrast, solution treatment at 1000 °C induced a high entropy effect [39], which made the alloy at an higher configurational entropy (ΔS_conf_) state to reduce the free energy of the alloy. The high ΔS_conf_ state was associated with higher lattice distortion and thus larger internal stress. It is noted that since the homogenization and solution treatments were conducted at high temperatures of 950 °C and 1000 °C, respectively, the alloying atoms can diffuse at these temperatures, although the sluggish diffusion nature of HEA slows down the diffusion rate. As a consequence, quenching from 1000 °C preserved the high ΔS_conf_ state at high temperature. The lattice distortion increased the friction at the interface of the B2 parent phase and B19′ martensite during the martensitic transformation. Additionally, the severe lattice distortion also resulted in larger stored elastic energy in the materials after martensitic transformation, which led to decreases in the M_f_ and A_s_ temperatures [27]. The associated internal stress of the severe lattice distortion hindered the B2→B19′ transformation and thus resulted in the decrement of the M_f_ and A_s_ temperatures.

### 3.4. Shape Memory Effect (SME) and Pseudoelasticity (PE)

**Figure 5:**
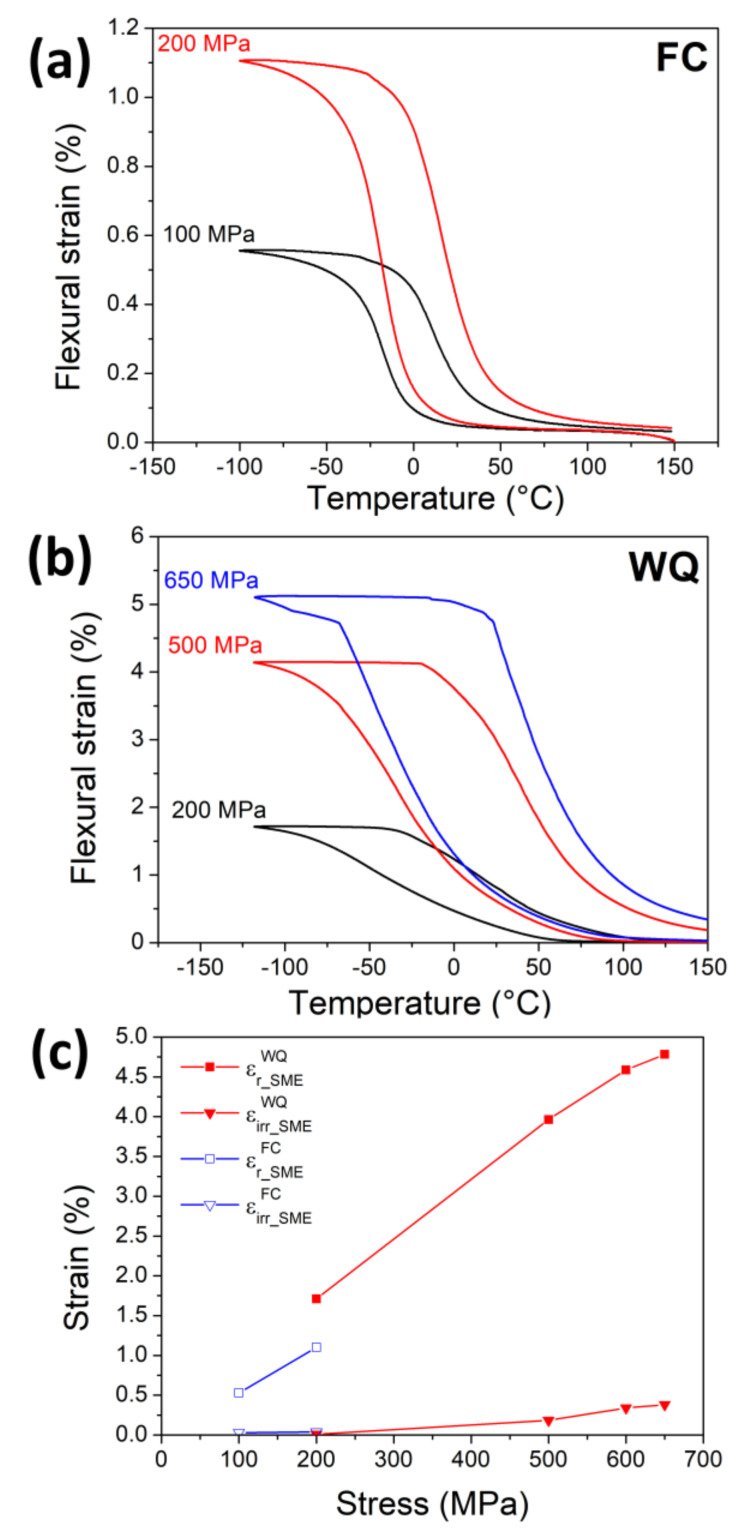
Shape memory effects of (**a**) FC and (**b**) WQ specimens with three-point bending test. (**c**) The recoverable and irrecoverable strains of the FC and WQ Ti_16.67_Zr_16.67_Hf_16.67_Ni_25_Co_10_Cu_15_ HESMAs. (Figure 5b is reprinted from Shape memory characteristics of (TiZrHf)_50_Ni_25_Co_10_Cu_15_ high entropy shape memory alloy, Vol. 162, Chih-Hsuan Chen and Yue-JinChen, Pages 185-189, Copyright (2019), with permission from Elsevier.)

The SMEs of the FC and WQ samples were tested by cooling and heating cycles under constant flexural stress with a three-point bending method. The sample was first heated to 150 °C to ensure that it was in a B2 parent phase. As shown in Figure 5a,b, the specimen showed flexural strain during cooling due to the formation of detwinned martensite. The subsequent heating process triggered the reverse transformation, so the flexural transformation strain could recover. Figure 5a shows that the FC sample exhibited a recoverable transformation strain ($\varepsilon_{r\_SME}^{FC}$) during the SME tests. The recoverable transformation strain is associated with the forward and reverse martensitic transformations. The flexural strains under 100 MPa and 200 MPa were 0.5% and 1.1%, respectively. It is noted that under the constant flexural stress of 300 MPa, the specimen fractured during phase transformation. The irrecoverable strains ($\varepsilon_{irr\_SME}^{FC}$) of the FC specimen, which was caused by permanent plastic deformation, remained smaller than 0.05% before the fracture occurred, indicating that the FC specimen was not able to tolerate plastic deformation.

Figure 5b shows the SME responses of the WQ sample under flexural stresses of 200, 500, and 650 MPa. It can be seen clearly from Figure 5b that the WQ sample sustained much higher flexural stresses. Additionally, under the flexural stress of 200 MPa, the WQ specimen exhibited a slightly higher transformation strain ($\varepsilon_{r\_SME}^{WQ}$) than that of the FC one ($\varepsilon_{r\_SME}^{FC}$). The $\varepsilon_{r\_SME}^{WQ}$ increased with increasing applied stress and then became saturated at about 600 MPa, followed by fracture at 700 MPa. At the same time, the irrecoverable strain of the WQ sample ($\varepsilon_{irr\_SME}^{WQ}$) increased with increasing applied stress, from 0.01% at 200 MPa to 0.38% at 650 MPa. It is also noted that the WQ specimen showed a wider temperature range during martensitic transformation, which is consistent with the DSC results shown in Figure 1.

Figure 5c summarizes the recoverable and irrecoverable strains of the FC and WQ samples. As can be seen from Figure 5c, the WQ sample sustained higher applied stress and also exhibited a higher transformation strain. This feature also meant that the FC sample was relatively brittle, so it fractured before all the martensite variants could reorientate and contribute to a larger transformation strain [40]. After solution treatment, the fracture stress of the WQ specimen was increased and the martensite variants were thus able to undergo a detwinning process, resulting in a much larger transformation strain.

**Figure 6:**
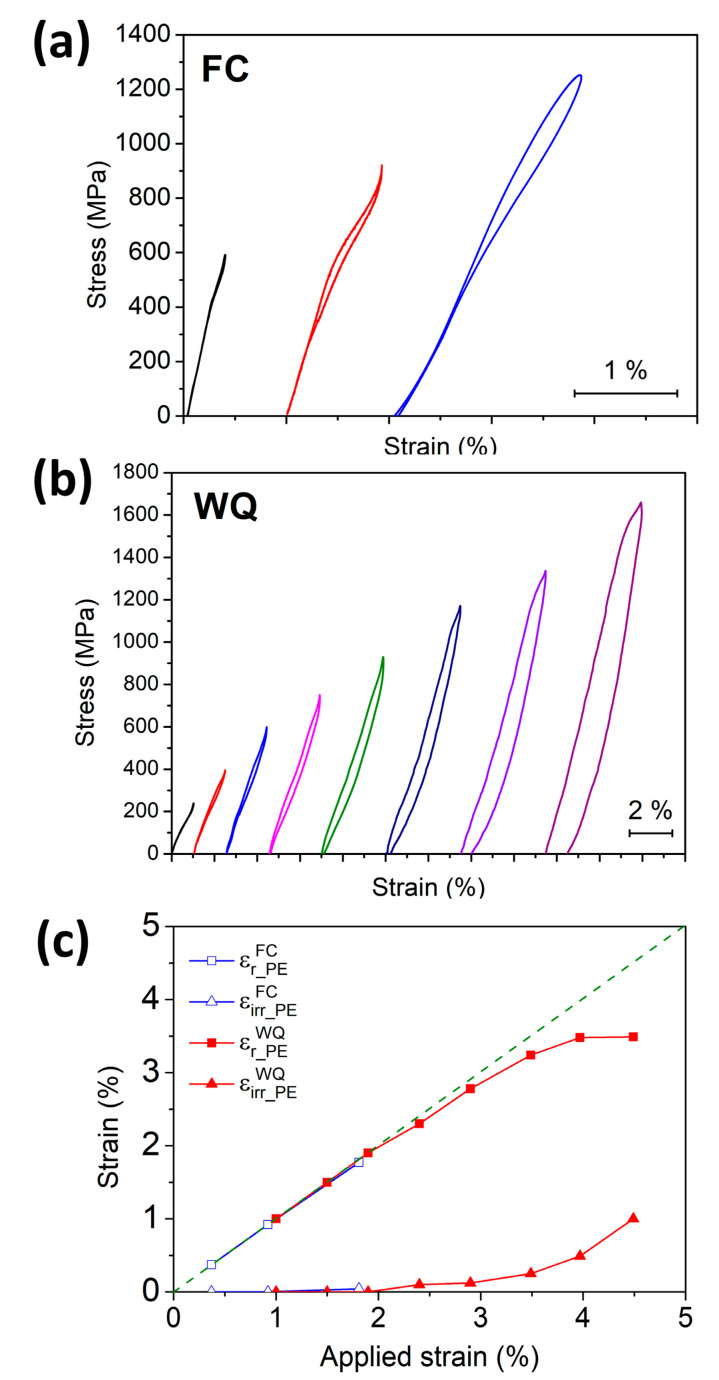
Pseudoelasticity of (**a**) FC and (**b**) WQ specimens under compression at 150 °C. (**c**) Summary of the recoverable and irrecoverable strains of the FC and WQ Ti_16.67_Zr_16.67_Hf_16.67_Ni_25_Co_10_Cu_15_ HESMAs. The green dotted line indicates 100% recovery of the applied strain.

Figure 6a,b shows the pseudoelasticity of the FC and WQ specimens under different applied strains. It is noted that transformation peaks of both samples showed a long tail upon heating, as can be seen in Figure 1. The temperature close to the intersection of the baseline and the tail of the heating peak for both samples is 150 °C. As a consequence, 150 °C was chosen as the testing temperature to ensure that both the FC and WQ samples were in a full austenite structure. From both Figure 6a,b, apparent stress hysteresis feature can be observed, which is a typical characteristic of pseudoelasticity in SMAs. Additionally, the deformation strains were recoverable. Both features indicate that martensitic transformation could be stress-induced in the FC and WQ samples and thus, they underwent pseudoelasticity at 150 °C.

Similarly to the SME results, the FC specimen showed nearly complete recoverability during the PE test with applied strain of up to about 1.8%, as shown in Figure 6a. When the applied strain was further increased to 3%, the specimen underwent brittle fracture. The WQ specimen also showed complete recoverability when the applied strain was below 2.0%, as shown in Figure 6b. However, the WQ specimen was able to sustain higher deformation strain, and it did not fracture when the applied strain was 4.5%. These features are consistent with the SME results, indicating that the HESMA was quite brittle and could not sustain plastic deformation in the FC condition. Figure 6c summarizes the recoverable strains ($\varepsilon_{r\_PE}^{FC}$ and $\varepsilon_{r\_PE}^{WQ}$) and irrecoverable strains ($\varepsilon_{irr\_PE}^{FC}$ and $\varepsilon_{irr\_PE}^{WQ}$) of the FC and WQ specimens as a function of applied strain during the compressive PE tests. As can be seen from Figure 6c, the values of $\varepsilon_{r\_PE}^{FC}$ and $\varepsilon_{r\_PE}^{WQ}$ were similar and showed complete recovery when the applied strain was smaller than 2%. However, the WQ specimen showed higher ductility and thus was able to sustain a higher applied strain. When the applied strain was increased from 2 to 4.5%, the $\varepsilon_{r\_PE}^{WQ}$ became saturated to 3.5%. At the same time, the $\varepsilon_{irr\_PE}^{WQ}$ increased to 1% when the applied strain reached 4.5%, indicating that plastic deformation was introduced during the PE test. It is noted that, compared with the WQ sample, 150 °C is a slightly high temperature for the FC sample to show pseudoelasticity due to its lower transformation temperatures. The FC sample may show slightly larger pseudoelastic strain at a lower testing temperature. The results shown in Figure 6 demonstrate that the WQ could sustain a much higher strength (did not fracture at 1700 MPa), and that the WQ specimen could undergo plastic deformation more readily than the FC one could.

The improvements in the SME and PE properties shown in Figure 5 and Figure 6 are considered to have originated from the partial dissolution of Ti_2_Ni-like second phase during the solution treatment process. It is well known that Ti_2_Ni-like second phase is a brittle intermetallic and is detrimental to the mechanical properties of TiNi-based SMAs [28,29]. In this HESMA, the solution treatment at 1000 °C enabled partial dissolution of some non-oxygen-stabilized Ti_2_Ni-like phase, and thus only the Ti_2_Ni-like phase that was stabilized by oxygen remained (as shown in Figure 3). The decrement in the volume fraction of the Ti_2_Ni-like phase reduced the brittleness of the HESMA. At the same time, the dissolution of the Ti_2_Ni-like phase slightly increased the (Ti, Zr, Hf) content in the matrix and thus caused increments of the M_s_ and A_f_ temperatures. Furthermore, the solution treatment increased the internal stress in the matrix, causing a broad transformation temperature range and improving the fracture stress of the alloy. Therefore, solution treatment of the Ti_16.67_Zr_16.67_Hf_16.67_Ni_25_Co_10_Cu_15_ HESMA is an effective way to improve its ductility and to prevent fracture during application, for it improves its shape memory functions.

## 4. Conclusions

In this study, the martensitic transformation behaviors, microstructure, composition, and functional properties of furnace-cooled (FC) and water-quenched (WQ) Ti_16.67_Zr_16.67_Hf_16.67_Ni_25_Co_10_Cu_15_ HESMAs were investigated and compared. Since the martensitic transformation is a diffusionless process, the sluggish diffusion effect in high entropy alloys did not hinder the martensitic transformation. Experimental results show that solution heat treatment at 1000 °C caused partial dissolution of the Ti_2_Ni-like second phase, which implies that the matrix showed higher thermal stability. The oxygen content in the remaining Ti_2_Ni-like second phase after solution treatment increased, indicating that oxygen significantly stabilized the precipitates such that they did not dissolve even at 1000 °C. The dissolution of the Ti_2_Ni-like second phase resulted in increases in the M_s_ and A_f_ temperatures due to increases in the (Ti, Zr, Hf) content in the B2 matrix. Additionally, the solution treatment induced a high entropy effect, which caused a higher degree of lattice distortion in the HESMA, and thus the decrement of the M_f_ and A_s_ temperatures and the broad transformation temperature range of the WQ sample. The partial dissolution of Ti_2_Ni-like second phase and the high entropy effect reduced the brittleness and strengthened the materials, leading to notable SME and PE performances, indicating that solution treatment is an effective way to improve the performance of the Ti_16.67_Zr_16.67_Hf_16.67_Ni_25_Co_10_Cu_15_ HESMA.

## Figures and Tables

**Table 1:** Chemical composition (at.%) of the B2 matrix and Ti_2_Ni-like phase in the FC and WQ Ti_16.67_Zr_16.67_Hf_16.67_Ni_25_Co_10_Cu_15_ HESMAs.

(at.%)	Ti	Zr	Hf	Ni	Co	Cu	O
**Furnace-Cooled (FC)**							
B2 matrix	16.6 ± 0.26	15.3 ± 0.21	17.0 ± 0.28	25.4 ± 0.21	10.5 ± 0.15	15.2 ± 0.16	-
(TiZrHf)_48.9_(NiCoCu)_51.1_							
Ti_2_Ni-like	29.3 ± 1.58	24.8 ± 1.47	11.7 ± 1.92	18.4 ± 0.65	7.5 ± 0.42	8.0 ± 0.57	0.34 ± 0.05
(TiZrHf)_65.8_(NiCoCu)_33.9_O_0.3_							
**Water-Quenched (WQ)**							
B2 Matrix	18.0 ± 0.17	15.3 ± 0.18	16.1 ± 0.33	25.1 ± 0.15	10.4 ± 0.09	15.1 ± 0.21	-
(TiZrHf)_49.4_(NiCoCu)_50.6_							
Ti_2_Ni-like	26.7 ± 0.71	22.2 ± 0.50	11.6 ± 0.76	17.3 ± 0.14	7.0 ± 0.31	7.1 ± 0.31	8.1 ± 0.43
(TiZrHf)_60.5_(NiCoCu)_31.4_O_8.1_							

**Table 2:** Area fraction of Ti_2_Ni-like phase estimated by SEM images.

	FC	WQ
Area fraction of Ti_2_Ni-like phase	2.2 ± 0.18%	1.5 ± 0.29%
